# Supplementary material for: Patient, family member, and health care provider perspective on barriers and facilitators to diabetic retinopathy screening in Thailand: A qualitative study
Source: PLoS One. 2023 Aug 3;18(8):e0289618. doi: 10.1371/journal.pone.0289618 (PMC10399890; doi:10.1371/journal.pone.0289618)
Supplement: S1 Text — (DOCX) [file pone.0289618.s001.docx]

**S1 Text**

**Focus group agenda**

**Patient**

- How do you know that you have DM?

*(Probe: Symptoms of diabetes mellitus and how can you know whether you have it?)*

- What did you plan after DM diagnosis?

*(Probe: Life style modifications like healthy diet, exercise, health check-up, management)*

- How many year did you have DM? Can you describe about the effect of DM on your health status?

*(Probe: consequences of diabetes, importance of blood sugar control)*

- Who did teach you about DM?

*(Probe: Source of information, type of information, material provided, usefulness of information)*

- What is your health status now?

*(Probe: Health problem caused by DM, feeling towards health, medications taken)*

- What do you know about DR?

*(Probe: Information provided to him/her, steps taken to safeguard the eyes)*

- When did you have last DR screening?

*(Probe: whether he/she attend screening, frequency of screening, challenges faced)*

**Family caregiver**

- What impact does DM have on your family's situation?

*(Probe: people in family affected, feeling after diagnosed as diabetes, impact of diabetes)*

- Are you confident that patient follows physician’s advice?

*(Probe: compliance to treatment, nature of advice given, medications, diet, exercise)*

- What obstacle did you face with when you take care of patient?

*(Probe: Difficulties on supervision, way of dealing, barriers faced in day to day life)*

- What do you know about effect of DM on patients’ health status?

*(Probe: complications of DM, treatments availability)*

- Who did teach you about DM?

*(Probe: Source of information, reading material, nature of information)*

- Does patient have any health problem caused by DM?

*(Probe: Co-morbidities of DM, vision related problems, preventive measures)*

- What do you know about DR?

*(Probe: understanding about DR, information provided, its stages of complications, symptoms)*

- When did patient have last DR screening?

*(Probe: Frequency of screening, understanding from patient side, compliance to screening, difficulties if any)*

- Do you have any suggestions from healthcare provider?

*(Probe: what will benefit the patient, education, travel allowance)*

**Healthcare provider**

- Who is involve in DR screening in your hospital?

*(Probe: Physicians/Ophthalmologists/optometrist/technicians)*

- Do you provide any prevention and knowledge to patient?

*(Probe: Health promotion, supportive services, education)*

- Do you have any DR prevention and screening?

*(Probe: Screening for other co-morbidities, Life style modification, diet)*

- What information do you require from patient?

*(Probe: History about health and ocular status, control of diabetes)*

- Do patients understand about DR and DM?

*(Probe: Level of understanding, compliance, feeling about DM and DR)*

How do you manage data of DM patients?

*(Probe: Frequency of follow-up, compliance to treatment, history of previous examination)*

- Who take care for the management of treatment cost?

*(Probe: From patient’s pocket/NGOs/Government fund/Insurance/trustee)*

- What do you think about patients, follow-up rate?

*(Probe: Reported only when they have symptoms/ as per adviced/compliance to follow-up)*

- Why do patients have poor compliance?

*(Probe: Reasons, not willing, long waiting time, financial issue, personal work, lack of awareness)*

- What can improve patient’s adherence?

*(Probe: Free consultation, travel allowance, patient education)*
