# Supplementary material for: Patient, family member, and health care provider perspective on barriers and facilitators to diabetic retinopathy screening in Thailand: A qualitative study
Source: PLoS One. 2023 Aug 3;18(8):e0289618. doi: 10.1371/journal.pone.0289618 (PMC10399890; doi:10.1371/journal.pone.0289618)
Supplement: Attachment — Submitted filename: Response to Reviewers.docx [file pone.0289618.s002.docx]

PLOS ONE

Journal Requirements:

When submitting your revision, we need you to address these additional requirements.

1. Please ensure that your manuscript meets PLOS ONE's style requirements, including those for file naming. The PLOS ONE style templates can be found at 
[https://journals.plos.org/plosone/s/file?id=wjVg/PLOSOne_formatting_sample_main_body.pdf](https://journals.plos.org/plosone/s/file?id=wjVg/PLOSOne_formatting_sample_main_body.pdf#inbox/_blank) and 
[https://journals.plos.org/plosone/s/file?id=ba62/PLOSOne_formatting_sample_title_authors_affiliations.pdf](https://journals.plos.org/plosone/s/file?id=ba62/PLOSOne_formatting_sample_title_authors_affiliations.pdf#inbox/_blank)

**Response:** The style and templates have changes as per suggested.

2. Please include a complete copy of PLOS’ questionnaire on inclusivity in global research in your revised manuscript. Our policy for research in this area aims to improve transparency in the reporting of research performed outside of researchers’ own country or community. The policy applies to researchers who have travelled to a different country to conduct research, research with Indigenous populations or their lands, and research on cultural artefacts. The questionnaire can also be requested at the journal’s discretion for any other submissions, even if these conditions are not met.  Please find more information on the policy and a link to download a blank copy of the questionnaire here: [https://journals.plos.org/plosone/s/best-practices-in-research-reporting](https://journals.plos.org/plosone/s/best-practices-in-research-reporting#inbox/_blank). Please upload a completed version of your questionnaire as Supporting Information when you resubmit your manuscript.

**Response:** Have added the supporting information.

3. Thank you for stating the following financial disclosure: 
"No"

At this time, please address the following queries:

a) Please clarify the sources of funding (financial or material support) for your study. List the grants or organizations that supported your study, including funding received from your institution.
   b) State what role the funders took in the study. If the funders had no role in your study, please state: “The funders had no role in study design, data collection and analysis, decision to publish, or preparation of the manuscript.”
   c) If any authors received a salary from any of your funders, please state which authors and which funders.
   d) If you did not receive any funding for this study, please state: “The authors received no specific funding for this work.”

**Response:** This study was supported by Lions Club International Foundation. SFP2050/UND. The funders had no role in study design, data collection and analysis, decision to publish, or preparation of the manuscript.

Please include your amended statements within your cover letter; we will change the online submission form on your behalf.

**Response:** Amended statement has been included in the cover letter.

4. Thank you for stating the following in the Acknowledgments Section of your manuscript: 
"This study was supported by Lions Club International Foundation. SFP2050/UND. The authors thank Dr. Robin Driscoll for administration support."

We note that you have provided funding information that is not currently declared in your Funding Statement. However, funding information should not appear in the Acknowledgments section or other areas of your manuscript. We will only publish funding information present in the Funding Statement section of the online submission form. 
Please remove any funding-related text from the manuscript and let us know how you would like to update your Funding Statement. Currently, your Funding Statement reads as follows:

**Response:** ‘This study was supported by Lions Club International Foundation. SFP2050/UND. The funders had no role in study design, data collection and analysis, decision to publish, or preparation of the manuscript’.

Please include your amended statements within your cover letter; we will change the online submission form on your behalf.

**Response:** Amended statement has been now added in the cover letter. Thank you!

[Note: HTML markup is below. Please do not edit.]

Reviewers' comments:

Reviewer's Responses to Questions

**Comments to the Author**

1. Is the manuscript technically sound, and do the data support the conclusions?

The manuscript must describe a technically sound piece of scientific research with data that supports the conclusions. Experiments must have been conducted rigorously, with appropriate controls, replication, and sample sizes. The conclusions must be drawn appropriately based on the data presented.

Reviewer #1: Partly

Reviewer #2: Partly

**Response:** Please see our further responses to reviewer’s comments, below.

2. Has the statistical analysis been performed appropriately and rigorously?

Reviewer #1: No

Reviewer #2: N/A

**Response:** Please see our further responses to reviewer’s comments, below.

3. Have the authors made all data underlying the findings in their manuscript fully available?

The [PLOS Data policy](http://www.plosone.org/static/policies.action#inbox/_blank) requires authors to make all data underlying the findings described in their manuscript fully available without restriction, with rare exception (please refer to the Data Availability Statement in the manuscript PDF file). The data should be provided as part of the manuscript or its supporting information, or deposited to a public repository. For example, in addition to summary statistics, the data points behind means, medians and variance measures should be available. If there are restrictions on publicly sharing data—e.g. participant privacy or use of data from a third party—those must be specified.

Reviewer #1: No

Reviewer #2: No

**Response:** All the data are provided in the manuscript. Data such as summary statistics, the data points behind means, medians and variance measures are not relevant given the qualitative nature of this study.

4. Is the manuscript presented in an intelligible fashion and written in standard English?

PLOS ONE does not copyedit accepted manuscripts, so the language in submitted articles must be clear, correct, and unambiguous. Any typographical or grammatical errors should be corrected at revision, so please note any specific errors here.

Reviewer #1: Yes

Reviewer #2: No

**Response:** We have tried our best to improve the language in the revised manuscript.

5. Review Comments to the Author

Please use the space provided to explain your answers to the questions above. You may also include additional comments for the author, including concerns about dual publication, research ethics, or publication ethics. (Please upload your review as an attachment if it exceeds 20,000 characters)

Reviewer #1: 1. Qualitative research required qualify person to be the interviewer or conduct the FGD, please submit CVs of the interviewers to ensure scientific soundness of this articles.

**Response:** Dr.Shuba Kumar (SK) is a social scientist and founder member of Samarth, an NGO engaged in social science research and training. She is trained in epidemiology and social science, has expertise in research methodology, particularly qualitative research methods and questionnaire validation. SK who is trained in qualitative research conducted a three day training workshop on qualitative research methods which was attended by three of the research team (PC, GK and SV). They observed SK conducting interviews and also carried out mock interviews/FGDs using the study guides. Feedback was provided to them to further improve their techniques following which they commenced doing the FGD. The duration of FGDs varied from 45 to 60 minutes. Under the PC supervision the other authors VN, WP and MT conducted the FGD. The CV of PC has been attached along with his workshop participation certificate.

2. Could the authors show the numbers of each participants from each hospital to ensure that the data is not mainly come from one hospital which may not reflect the context of the country?

**Response:** Number of participants from each hospital are listed below.

| *Participants* | *Hospital 1 (Srisangwal Hospital in Mae Hong Sorn province)* | *Hospital 2 (Pakchongnana Hospital in Nakornrajasima province)* | *Hospital 3 (Wiengsra Crown Prince Hospital in Surat Thani province)* | *Hospital 4 (Rajavithi Hospital in Bangkok)* | *Hospital 5 (Sam Roi Yot Hospital in Prachuap Khiri Khan province)* |
| --- | --- | --- | --- | --- | --- |
| *Patients* | *9* | *12* | *5* | *11* | *10* |
| *Family members* | *11* | *8* | *6* | *6* | *10* |
| *Health care providers* | *6* | *6* | *7* | *7* | *8* |

3. This study exclude patients with no light perception which is the appropriate target group to gain the information about the barrier to access to the health care so the author should discuss this issue in the part of discussion.

**Response:** Thank you. We have discussed this as following in line number 419-423

Although patients with no light perception are the appropriate group to gain information about the barrier, there are obstacles faced by the visually impaired patients due to lack of nonverbal signals such as hand gestures, nods, eye contacts and facial expression in the FGD. A study by Qui et al. reported having unpleasant feelings during the conversation as a result of not being able to read nonverbal cues such hand gestures, nods, eye contact, and facial expressions during face-to-face communication. To completely understand the challenges experienced in receiving diabetic care, future studies must concentrate on individuals who are visually impaired.

4. Please provide the detail of how to sampling the individual key informants because the selection bias might be occurred which can reduce the possibility of generalizability of the study.

**Response:** The selection of patients was done purposively using maximum variation sampling. We looked at the retinal specialists' appointment schedules with a focus on the obstacles to DR care. We would visit the outpatient department of the particular retinal expert on the day of the appointment as scheduled. We recruited men and women aged 18 years or more with diabetes at least for 5 years or more belonging to different age, education and occupation. Selection of a varied group of participants enabled us to obtain diverse perceptions on the study topic which was to understand, perceptions and awareness about DR, the need for regular eye screening, perceived barriers faced in seeking eye care, in addition to awareness about diabetes and diabetic related complications. Being a qualitative study, generalizations are not possible as the samples are typically small and purposively selected but they enable deeper insights and understanding. This has been now discussed in line number 110-113.

Reviewer #2: Dear authors,
Thank you so much for an opportunity to review this manuscript. It is a good study exploring barriers of DR screening as the uptake of the service is apparently low. However, I think the results need to be restructured because the current version does not seem to exhibit barriers and factors influencing compliance to the service. My major and specific comments describe below:
Major comments.
1. The current themes do not represent barriers and facilitators of the DR screening. The current results are predominantly related to awareness/knowledge/behaviors of diabetes/care/DR. However, there is no connection between these findings and the service uptake. This needs to explain more how they affect the service use.

**Response:** Thank you for the suggestion. We have now included the following section explaining the connection between our findings and the service uptake in Page number 8-17

2. There is only one theme ‘Barriers to DR screening’ which is directly related to the objective. While the other themes are likely a background information and situation of patients and care. This is why authors need to restructure the results.

**Response:** We have now reconstructed the result part.

3. Authors currently structure the results by views of different stakeholders. Would it be better to combine the same theme into one and explain it from each perspective? For example: ‘the theme ‘care-seeking practice’ is emerged by all stakeholders, can you combine them into one and expand a little bit more why this impact to the screening uptake.

**Response:** Thank you for this comment to modify. We have now re-structured the result as following to include your suggestion in page number 10-11.

4. When extracting the data, authors may concern barriers related to service delivery such as human resources, finance, information system, policy, etc. to cover all the barriers comprehensively.

**Response:** Thank you. These have been now discussed as following in Page number 14-15.

Barriers to DR screening included a lack of availability of ophthalmologists, long waiting times, financial constraints, transportation problems, the absence of accompanying individuals to attend appointment, and ignorance of DR screening. These challenges were cited by patients as reasons for not going to the hospital. Other reasons including long travel time and forgetting the appointment date.

5. Quotations supported for each theme are too many, please be selective. Use only ones that outstanding.

**Response:** Selective quotes are now added.

6. I do not quite agree by reporting the percentage of participants by gender or by opinion. This is a qualitative study using FGD. Opinions obtained from FGD are ‘Group’s opinion’, not the opinion of each individual. Although there are some qualitative researchers do that but I think it is not appropriate to show how many percent agree/disagree. Instead, authors should demonstrate all the issues raised by all groups either positive or negative.

**Response:** We agree with your suggestion and we have modified the contents in the result part accordingly.

7. I must say there are many sentences that are not understandable. I think this manuscript also need an extensive English proof reading.

**Response:** We have tried our best to improve English and simplify the sentences in the revised manuscript.

Specific comments.
Abstract. This need to be revised after the manuscript revision completed.

**Response:** This has been updated as per the revisions.

Introduction.
Line 75: How many diabetic patients have been screened for DR recently? This information should be stated.

**Response:** This has been stated in line number 74-75.

Methods:
Line 93-98: Please blind the hospital names and describe them by their characters e.g. hospital type, size, location, etc.

**Response:** Thank you for the suggestion. We have modified this to address your comment. (line number 97-99)

Line 113: As the authors use FGD, the instrument is rather ‘a focus group agenda’, not an interview guide.

**Response:** Thank you! We have revised the manuscript as you suggested (line number 115)

Comment: Recruitment of health care providers is missed, this should be stated.

**Response:** This has been now stated as following in the revised manuscript (line number 116-118)

Line 116-121: I guess here is a list of questions used in the FGD. These sentences are not completed, some verbs are missed. It might be better to illustrate by table.

**Response:** Have re-framed the lines now. (119-123)

Line 146-146: Was the coding text performed? As authors set up some categories prior to analyzing the data, where do these categories come from? Was there any theoretical framework used for data analysis?

**Response:** We did use the framework analytical approach which is also termed as thematic analysis or content analysis. Furthermore, our research included young researchers who were not highly experienced in qualitative data analysis. The framework approach which involves a structured and systematic method of organising our data into spreadsheets was something they could understand and undertake. (Line number 143-148)

Line 151: … thereby setting the stage for 151 theme development … I am not sure what this means?

**Response:** This was a typo. This sentence has been revised as following to add clarity line number (151-153)

Line 208-209: Please clarify more how ‘changing hospital’ is related to self-ownership. And How does this link to barriers of the screening.

**Response:** Generally when the glucose level is not get controlled patients may have a tendency to visit another hospital for better care. Have rephrased the line. (line number:181-183)

Line 240-241: ‘ophthalmologists are not responsible for any blindness or complications’ … Is this message a routine practice?

**Response:** It is not used in a routine practice, but the message focuses on the risks that were raised, such as the type and effects of the treatment, the results of the surgery, or the patient concerns around the disease prognosis. The line has rephrased now in the line number 252-257.

Line 261-263: Barriers for attendance for DR screening …. Do the patients also acknowledge these barriers?

**Response:** Yes, these were the barriers reported by the patient during the FGD (line number:302-306)

Line 265-266: ‘Doctors-led diabetes management groups to discuss and solve the queries raised by patients were considered an important element in the follow-up of patients’ I am not sure what this means?

**Response:** This sentence has been revised as following in line number 338-339

‘People preferred a discussion group with doctors to address their concerns and questions about managing their diabetes and encourage compliance.’

Line 269-270: ‘Patients thought a day dedicated to DR screening only would be of benefit.’ I am not sure what this means?

**Response:** This sentence has been revised as following to add clarity in line 341-342

For the theme ‘Care-seeking practice…’ I think the better term to use is ‘behavior’ as I think authors intend to explain behaviour of patients how they seek care.

**Response:** Thank you! We have renamed the theme as per your suggestion (Page number 10)

Discussion

Line 458: Knowing the difference between glaucoma and DR is rather a knowledge, not a health literacy.

**Response:** Thank you! We have revised this as per your suggestion (line number 402-403)

The discussion points should also mention issues related to service delivery such as human resources, finance, information systems, or screening policy.

**Response:** These points have been now discussed in line number 436-438 in the revised manuscript.

Conclusion need to be revised. The current one does not reflect to the objectives.

**Response:** We have revised the conclusion as following to reflect to the objectives. Line number 453-457.

‘The majority of patients were passive and had great faith in the HCPs. Family participation in DM care can improve health-related behaviors in patients. The involvement of non-physician providers working in the field of diabetes has a significant impact in encouraging patients to attend DR screening. Our findings may be useful in designing policies to make DR screening in Thailand more accessible’

6. PLOS authors have the option to publish the peer review history of their article ([what does this mean?](https://journals.plos.org/plosone/s/editorial-and-peer-review-process#inbox/_blank)). If published, this will include your full peer review and any attached files.

If you choose “no”, your identity will remain anonymous but your review may still be made public.

**Do you want your identity to be public for this peer review?** For information about this choice, including consent withdrawal, please see our [Privacy Policy](https://www.plos.org/privacy-policy#inbox/_blank).

Reviewer #1: No

Reviewer #2: No
