# Supplementary material for: Patient, family member, and health care provider perspective on barriers and facilitators to diabetic retinopathy screening in Thailand: A qualitative study
Source: PLoS One. 2023 Aug 3;18(8):e0289618. doi: 10.1371/journal.pone.0289618 (PMC10399890; doi:10.1371/journal.pone.0289618)
Supplement: Attachment — Submitted filename: Response to Reviewer.doc [file pone.0289618.s003.doc]

Journal Requirements:

Please review your reference list to ensure that it is complete and correct. If you have cited papers that have been retracted, please include the rationale for doing so in the manuscript text, or remove these references and replace them with relevant current references. Any changes to the reference list should be mentioned in the rebuttal letter that accompanies your revised manuscript. If you need to cite a retracted article, indicate the article’s retracted status in the References list and also include a citation and full reference for the retraction notice.

***Response:*** *We have reviewed the reference list as per suggested.*

[Note: HTML markup is below. Please do not edit.]

Reviewers' comments:

Reviewer's Responses to Questions

**Comments to the Author**

1. If the authors have adequately addressed your comments raised in a previous round of review and you feel that this manuscript is now acceptable for publication, you may indicate that here to bypass the “Comments to the Author” section, enter your conflict of interest statement in the “Confidential to Editor” section, and submit your "Accept" recommendation.

Reviewer #1: All comments have been addressed

Reviewer #2: All comments have been addressed

***Response:*** *Thank you for your review and insightful comments. We found them very useful in improving our manuscript.*

2. Is the manuscript technically sound, and do the data support the conclusions?

The manuscript must describe a technically sound piece of scientific research with data that supports the conclusions. Experiments must have been conducted rigorously, with appropriate controls, replication, and sample sizes. The conclusions must be drawn appropriately based on the data presented.

Reviewer #1: Yes

Reviewer #2: Partly

***Response:*** *We have further revised the manuscript to make it technically sound better, wherein data directly support the conclusion. Please see our ‘further responses to reviewer’s comments’, below.*

3. Has the statistical analysis been performed appropriately and rigorously?

Reviewer #1: N/A

Reviewer #2: N/A

***Response:*** *Thank you.*

4. Have the authors made all data underlying the findings in their manuscript fully available?

The [PLOS Data policy](http://www.plosone.org/static/policies.action#sharing) requires authors to make all data underlying the findings described in their manuscript fully available without restriction, with rare exception (please refer to the Data Availability Statement in the manuscript PDF file). The data should be provided as part of the manuscript or its supporting information, or deposited to a public repository. For example, in addition to summary statistics, the data points behind means, medians and variance measures should be available. If there are restrictions on publicly sharing data—e.g. participant privacy or use of data from a third party—those must be specified.

Reviewer #1: Yes

Reviewer #2: No

***Response:*** *All the data are provided in the results and the supplementary materials. The findings will be also deposited in Anglia Ruskin Research Online (ARRO). We have tried our best to cover all the findings in the revised manuscript.*

5. Is the manuscript presented in an intelligible fashion and written in standard English?

PLOS ONE does not copyedit accepted manuscripts, so the language in submitted articles must be clear, correct, and unambiguous. Any typographical or grammatical errors should be corrected at revision, so please note any specific errors here.

Reviewer #1: Yes

Reviewer #2: No

***Response:*** *We have fixed the typos and tried our best to improve the language in the revised manuscript.*

6. Review Comments to the Author

Please use the space provided to explain your answers to the questions above. You may also include additional comments for the author, including concerns about dual publication, research ethics, or publication ethics. (Please upload your review as an attachment if it exceeds 20,000 characters)

Reviewer #1: (No Response)

Reviewer #2: Dear authors
Thank you for the revised manuscript which has been improved significantly. However, I still have two points which I think it may not be the case:
1. Discussion and conclusion about a need of non-physician clinician involvement, as I do not see this finding mentioned in the result section yet. So, this point of discussion and conclusion is not aligned to the findings. Please check and revise.

***Response:*** *Thank you for highlighting this important point. Apologies for the missing point, we have included non-physician clinician involvement throughout the ‘Results’ section.*

*‘Family members and, non-physician clinicians (such as diabetes nurses, diabetes educators, physician assistants) who work in the field of diabetes play a vital role in encouraging patients to attend diabetes and DR follow-ups visits regularly (Abstract, page 3).*

*‘Therefore physician and, non-physician clinicians felt that patients should be given a guidebook, posters, books, videos, or brochures with the necessary information reminding them about the possible complications of uncontrolled diabetes in eyes, feet, etc. (Page 11, last paragraph).*

‘*Patients who are unable to travel to the hospital due to distance, financial constraints, or other factors should be screened at a primary care center near their home with the help of non-physician clinicians.’ (Page 16, last paragraph).*

*‘One caregiver identified social stigma, for example, poorer patients felt ignored by the hospital, and suggested that education should be provided by non-physician clinicians (nurse/nurse aid) to poor and elderly patients.’(Page 16, last paragraph)*

*‘The involvement of non-physician clinicians working in the field of diabetes has a significant impact on encouraging patients to attend DR screening (Page 20, Conclusion section).*

2. The point regarding equity of health insurance scheme (line 372-375, 440-441) since the authors have stated in line 323 “Despite screening service for diabetes being free...” which I think the DR screening is included in the routine service for diabetic patients and covered all insurance scheme. Is this correct? If so, then this point may not be the case. Please check and revise.

***Response:****. Thank you. The point have been re-phrased as “Despite screening service for diabetes being covered by insurance scheme” in the revised manuscript. (Page number 15, line number 320)*

Abstract
Line 51-52: is this matched to the findings described in maintext?

***Response:*** *Thank you we have added the relevant text now (Line number 51-52)*

Line 55-59: Same as point 1 & 2 above.

***Response:*** *We have re-phrased the line now (Line number 55-57)*

Grammatic errors and other little things
Line 319: … did not adopted …

***Response:*** *Thank you! This was a typo and has been now corrected. (Line number 390)*

Line 345: … that that they could ….

***Response:*** *Thank you! This was a typo and has been now corrected. (Line number 342)*

Line 346: … We have a Line group …. An explanation in brackets of “Line” is needed e.g. a chat application popularly used in Thailand or something like this.
Kind regards

***Response:*** *Line group is a freeware application in iOS, android and window software. Instant communication by text, photographs, and voice can be created. The line group comprising many people can be used to communicate in a group. Only invitees can enter to line group. This has been mentioned in the revised manuscript in Page 16, line number 344-345.*

*‘“We have a “Line group” (freeware application software to communicate with many patients) for diabetic patients as well, in which we can change our appointment date within 7 days and also consult for the medications. (FGD 5)”*

7. PLOS authors have the option to publish the peer review history of their article ([what does this mean?](https://journals.plos.org/plosone/s/editorial-and-peer-review-process#loc-peer-review-history)). If published, this will include your full peer review and any attached files.


If you choose “no”, your identity will remain anonymous but your review may still be made public.


**Do you want your identity to be public for this peer review?** For information about this choice, including consent withdrawal, please see our [Privacy Policy](https://www.plos.org/privacy-policy).

Reviewer #1: No

Reviewer #2: No

***Response:*** *Thank you.*

[NOTE: If reviewer comments were submitted as an attachment file, they will be attached to this email and accessible via the submission site. Please log into your account, locate the manuscript record, and check for the action link "View Attachments". If this link does not appear, there are no attachment files.]

While revising your submission, please upload your figure files to the Preflight Analysis and Conversion Engine (PACE) digital diagnostic tool, <https://pacev2.apexcovantage.com/>. PACE helps ensure that figures meet PLOS requirements. To use PACE, you must first register as a user. Registration is free. Then, login and navigate to the UPLOAD tab, where you will find detailed instructions on how to use the tool. If you encounter any issues or have any questions when using PACE, please email PLOS at [figures@plos.org](mailto:figures@plos.org). Please note that Supporting Information files do not need this step.
